# Impact of Yuanjiang *Miscanthus lutarioriparius* Aqueous Extract on Texture, Flavor Profile, and Antioxidant Activity of Yogurt During Storage

**DOI:** 10.3390/molecules30204042

**Published:** 2025-10-10

**Authors:** Siyi He, Jianglin Wang, Xia Tang, Xiankang Fan, Jie Luo, Tong He, Hui Zhou

**Affiliations:** 1 College of Food Science and Technology, Hunan Agricultural University, Changsha 410128, China; siyihe0041@163.com (S.H.); 17363897816@163.com (J.W.); fanxiankang2022@163.com (X.F.); luojie@hunau.edu.cn (J.L.); 2 Royal Group Hunan Ushi Dairy Co., Ltd., Changsha 410699, China; 19217403413@163.com

**Keywords:** yogurt, volatile components, functional food

## Abstract

Yuanjiang *Miscanthus lutarioriparius*, which is rich in various bioactive components, exhibits significant potential in the development of functional foods. However, research on its application in dairy products remains relatively limited. This study fermented yogurt using different concentrations of Yuanjiang *Miscanthus lutarioriparius* water extract (0%, 0.1%, 0.2%, and 0.4%) as a functional additive, investigating its effects on the rheological properties, oxidative capacity, sensory quality, and volatile components of yogurt during storage. The results showed that during storage, the rheological properties (such as moisture content, apparent viscosity, storage modulus, etc.), the viable counts of *Streptococcus thermophilus* and *Lactobacillus bulgaricus*, and the DPPH/ABTS/FRAP radical scavenging rates of asparagus yogurt were significantly superior to those of the control group (*p* < 0.05), indicating that the lactic yogurt exhibited better texture, stability, and overall sensory acceptance. The 0.2% addition group exhibited the best inhibitory effect on lactic acid bacteria after acidification and the most stable acidity changes. The 0.4% addition group achieved an ABTS radical scavenging rate of 58.4% on the 7th day of storage, significantly higher than other groups (*p* < 0.05). The asparagus yogurt contained 64 volatile flavor compounds (20.31% alcohols and 21.88% ketones), which was higher than the control group (45 compounds), and introduced new aldehydes (tridecanal) and esters (methyl salicylate, ethyl palmitate), imparting a mild sourness and spicy flavor. Sensory evaluation results indicated that the 0.2% addition group scored the highest in texture, flavor, and taste, aligning with its rheological properties and color. This provides a theoretical basis for the development of highly stable and active functional asparagus yogurt.

## 1. Introduction

Fermented milk products, particularly yogurt, represent a cornerstone of functional foods, renowned for their nutritional density and profound associations with human health and wellness [1]. The fermentation process, mediated by probiotic cultures such as *Streptococcus thermophilus* and *Lactobacillus bulgaricus*, not only extends shelf-life but also enhances the bioavailability of nutrients and generates bioactive compounds [2]. Regular consumption of yogurt is linked to a reduced risk of gastrointestinal disorders, cardiovascular diseases, and certain cancers, attributed to its ability to modulate gut microbiota, lower serum cholesterol, and exert anti-mutagenic activities [3]. Beyond these health benefits, yogurt is a rich source of high-value proteins, essential vitamins, and minerals, presented in a highly digestible form [4]. In response to growing consumer demand for foods that support well-being, the dairy industry is increasingly focused on innovating beyond traditional formulations. This innovation targets the development of products with enhanced functional properties, such as reduced fat content and the incorporation of bioactive ingredients that offer targeted health advantages beyond basic nutrition [5].

A promising strategy for developing such novel functional foods involves the fortification with plant-based extracts rich in phytochemicals. These compounds, including polyphenols and flavonoids, can significantly augment the antioxidant capacity and other health-promoting properties of food matrices [6]. Yuanjiang *Miscanthus lutarioriparius* (also known as *Triarrhena lutarioriparia* shoot), a gramineous plant native to specific regions of China, emerges as an exceptionally potent source of these bioactives. Hailed as “Dongting Cordyceps” for its remarkable nutritional profile, its young shoots are abundant in dietary fiber, all 16 essential amino acids, vital minerals (particularly selenium at concentrations over ten times that of common vegetables), and a diverse array of flavonoids [7]. Flavonoids are particularly notable for their extensive pharmacological functions, which include neuroprotective, antihypertensive, anti-inflammatory, anti-tumor, and hypoglycemic effects [8]. Despite this immense potential, the application of Yuanjiang *Miscanthus lutarioriparius* in the food industry, and particularly in dairy products, remains largely unexplored, limiting the realization of its edible and medicinal value.

The integration of plant extracts into yogurt is not without its challenges, as it can profoundly influence the product’s physicochemical, sensory, and microbial characteristics. Successful incorporation requires a delicate balance to enhance functionality without compromising the desired texture, flavor, and stability that consumers expect. For instance, studies have demonstrated that algal extracts can enrich yogurt with omega-3 fatty acids, while fruit additions can be optimized to improve sensory appeal [9]. These examples underscore the potential of strategic fortification but also highlight the necessity of extract-specific characterization. The aqueous extract of Yuanjiang *Miscanthus lutarioriparius*, likely rich in polar flavonoids and phenolic acids, could interact with milk proteins (casein and whey), potentially altering the gelation process during fermentation, the rheology of the final product, and the viability of starter cultures throughout storage. Furthermore, its distinct volatile compounds could either complement or disrupt the complex flavor profile of yogurt, which is derived from a balance of acids, carbonyls, alcohols, and esters produced by bacterial metabolism and lipid oxidation [10,11]. A critical, unanswered question is how these interactions evolve during storage, a period marked by ongoing biochemical changes such as post-acidification and proteolysis, which can affect everything from antioxidant activity to microbial survival.

Consequently, an in-depth study is critically required to assess how Yuanjiang *Miscanthus lutarioriparius* aqueous extract influences the diverse quality aspects of yogurt over time. This research intends to fill the existing knowledge void by methodically adding the extract at different concentrations (0.1%, 0.2%, 0.4%) into a skim milk yogurt formulation. Our hypothesis posits that the extract will enhance the antioxidant capacity in a dose-dependent manner and improve the textural and rheological characteristics of yogurt by modifying the casein gel structure and promoting probiotic viability, all while contributing a distinct and appealing volatile flavor profile. To evaluate this, we meticulously measured key quality indicators—including titratable acidity, water-holding capacity, viscosity, viscoelastic properties (G′ and G″), probiotic viability, texture profile, color, and antioxidant activity (DPPH, ABTS, FRAP)—throughout a 28-day storage duration. Sophisticated GC-MS analysis was utilized to analyze the volatile compound profile, and consumer acceptance was assessed using structured sensory evaluation. The results of this research provide a significant theoretical basis for the high-value application of Yuanjiang *Miscanthus lutarioriparius* and open avenues for creating a new generation of stable, functional dairy products boasting improved health benefits and enhanced sensory features.

## 2. Results and Discussion

### 2.1. Changes in Titratable Acidity Yogurt Containing Aqueous Extract of Asparagus During Storage

**Figure 1:**
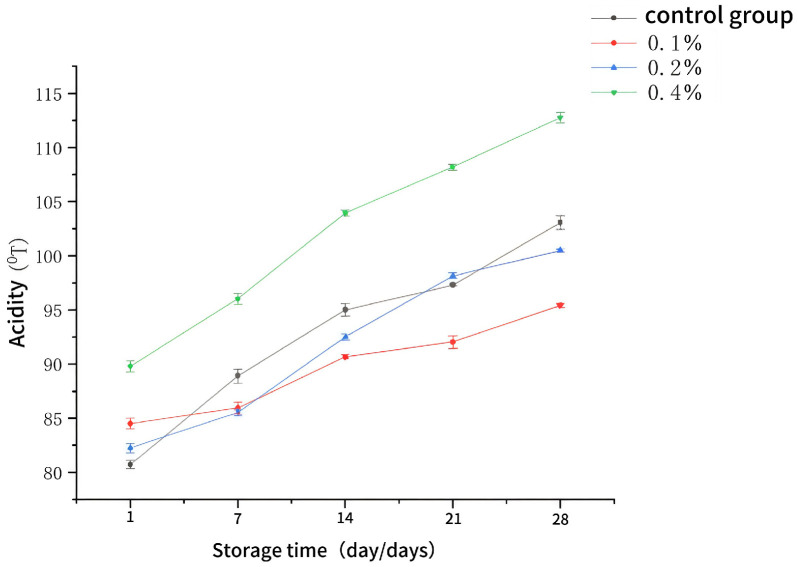
The change in acidity of yogurts during storage.

Acidity is one of the most fundamental quality characteristic indicators of fermented products. It can not only inhibit the growth of pathogenic microorganisms to ensure product safety but also affect the taste during the fermentation process [10,11]. As illustrated in Figure 1, the acidity values of both the control group and the experimental group (with the addition of aqueous extract of asparagus) increased along with the prolongation of storage time. During the storage period, the yogurt with a 0.4% addition level exhibited significantly higher acidity compared to the other groups (*p* < 0.05), indicating that a high concentration of asparagus aqueous extract led to severe post-storage acidification. When comparing the acidity trends between yogurt with a 0.2% addition level and that with a 0.1% addition level, the former demonstrated higher acidity and a relatively more stable post-acidification trend compared to the blank control group. This suggests that the quality of yogurt with a 0.2% addition level was also improved, and that a low concentration addition level could effectively inhibit post-acidification in yogurt, thereby ensuring product quality. The exogenous additives can provide nutrients for the growth of certain acid-producing microorganisms, thereby promoting acid synthesis and reducing acidity [12].

### 2.2. Changes in Viscosity of Yogurt Containing Aqueous Extract of Asparagus During Storage

**Figure 2:**
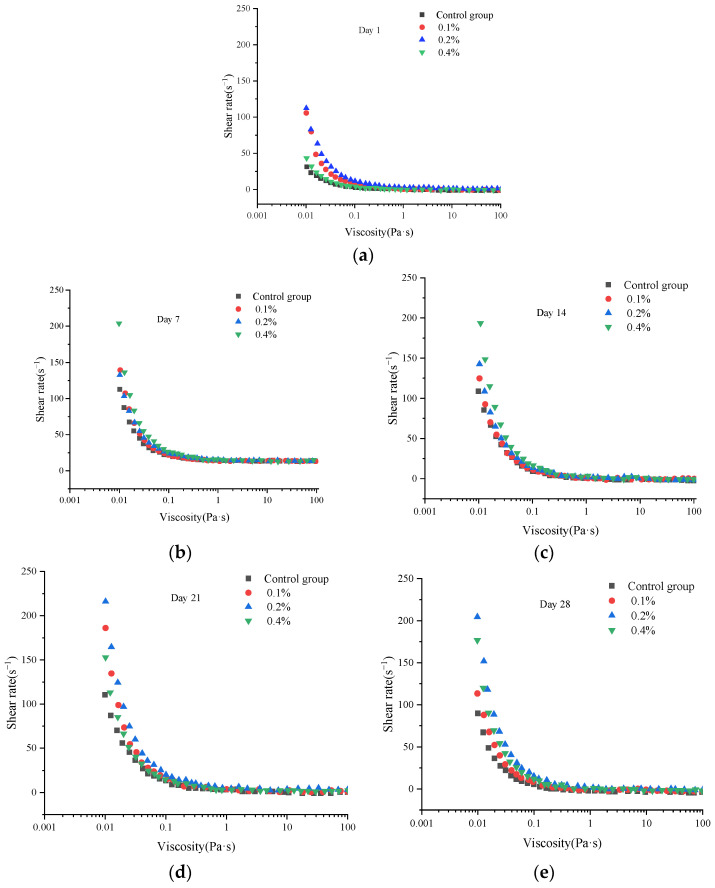
Changes in viscosity of yogurt during storage on (**a**) day 1, (**b**) 7, (**c**) 14, (**d**) 21, and (**e**) 24.

The viscosity of yogurt is its key rheological property, which directly determines the product’s physical stability, texture perception, and overall sensory quality by influencing the protein gel network structure, whey retention capacity, and oral lubrication characteristics [13,14]. As can be seen from Figure 2, during storage, the viscosity of both experimental groups and the control group increased. This phenomenon is likely attributable to the continued formation of the casein gel network structure during storage, thereby enhancing viscosity. By day 28 of storage, viscosity in both experimental and control groups decreased compared to days 7, 14, and 21. This reduction may result from the effects of post-acidification during the maturation phase.

Throughout the storage period, the viscosity of the experimental groups was significantly higher than that of the control group (*p* < 0.05). Notably, yogurt with a 0.2% asparagus aqueous extract addition exhibited significantly higher viscosity than both other experimental groups (0.1%, 0.4%) and the control group. This suggests that the 0.2% concentration may more effectively enhance the binding capacity with the casein gel network. These results collectively demonstrate that the addition of asparagus aqueous extract can effectively improve yogurt viscosity. Research on how specific components (such as extracellular polysaccharides EPS or proteins) can alter yogurt viscosity and further influence its oral texture perception and sensory scores [15].

### 2.3. Strain Sweep Results and Analysis

**Figure 3:**
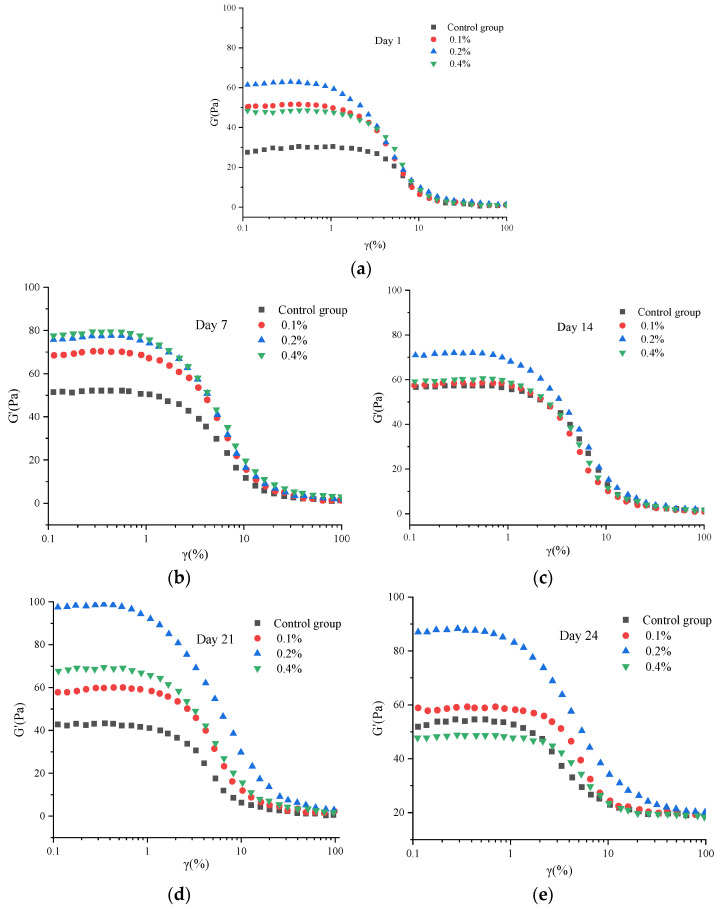
The viscosity of yogurts during storage on (**a**) day 1, (**b**) 7, (**c**) 14, (**d**) 21, and (**e**) 24.

The yogurt stress sweep test provides critical rheological evidence for evaluating the structural strength and stability of its gel network by defining parameters such as the linear viscoelastic region and the storage modulus (G′) [16,17]. As indicated in Figure 3, the linear viscoelastic region (LVR) spanned from 0.01% to 10%, with a critical strain value of 10%. Within the LVR, the (G′) remained constant and independent of external perturbations. The gel systems of experimental groups (supplemented with Yuanjiang *Miscanthus lutarioriparius* aqueous extract) exhibited higher G′ values than the blank control group. G′ demonstrated a consistent upward trend with increasing concentrations of asparagus extract, and the 0.2% addition group showed significantly greater G′ compared to other groups (*p* < 0.05). These results indicate that incorporating Yuanjiang *Miscanthus lutarioriparius* extract promotes the formation of a denser and more robust network structure in the polysaccharide-casein matrix.

### 2.4. Frequency Sweep Results and Analysis

**Figure 4:**
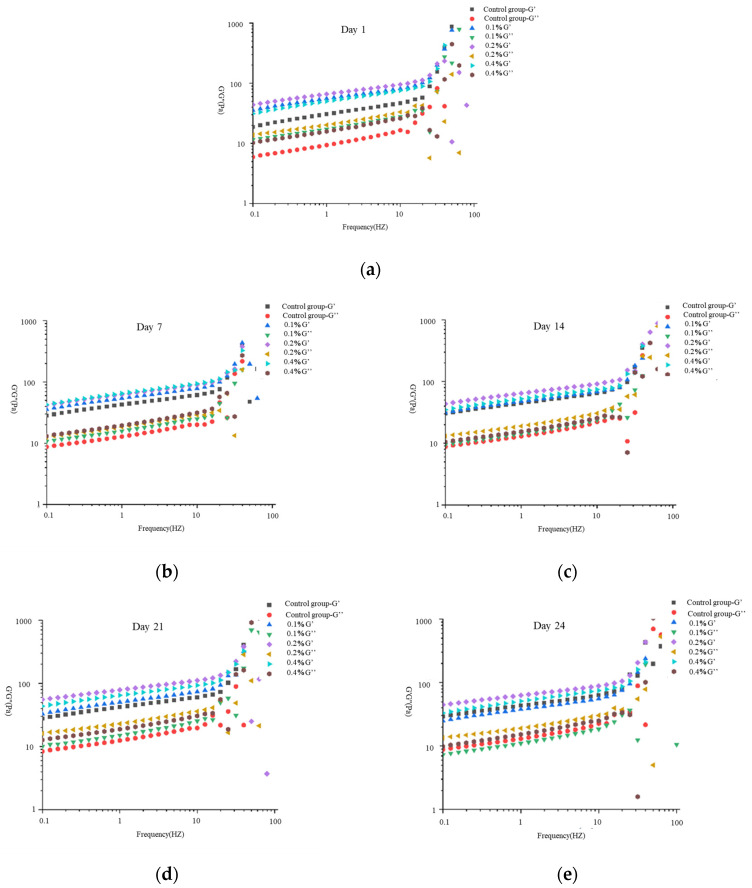
Changes in G′ and G″ in yogurts during storage on (**a**) day 1, (**b**) 7, (**c**) 14, (**d**) 21, and (**e**) 24.

Frequency sweep of yogurt characterizes the frequency dependence of storage modulus (G′) and Loss modulus (G″), revealing the relaxation characteristics and structural stability of its gel network at different time scales, providing critical rheological basis for predicting product shelf-life stability and oral processing behavior [18,19], as indicated in Figure 4. During the 1–28 day storage period, the 0.2% addition group consistently exhibited the highest G′ among all four yogurt formulations, indicating superior structural stability. Concurrently, the G′ and G″ values of the 0.4% addition group remained lower than those of the 0.2% group throughout storage, suggesting potential structural incompatibility between the high-concentration Yuanjiang *Miscanthus lutarioriparius* aqueous extract and the protein network. This incompatibility may account for the persistently lower viscoelastic moduli in the 0.4% group compared to the 0.2% formulation.

From day 1 to 21 of storage, the control group (without extract) demonstrated significantly lower G′ and G″ values than the experimental groups (*p* < 0.05). This systematic enhancement of viscoelastic parameters in extract-supplemented yogurts confirms that Yuanjiang *Miscanthus lutarioriparius* aqueous extract effectively enhances the structural stability of yogurt during extended storage.

### 2.5. Changes in Viable Bacterial Count of Asparagus Yogurt During Storage Period

**Figure 5:**
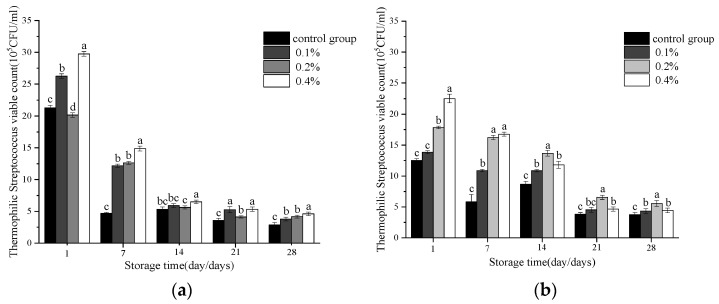
Changes in the cells number of (**a**) *S. thermophilus* and (**b**) *L. bulgaricus* during storage. Note: Different lowercase letters indicate significant differences (*p* < 0.05).

Probiotics have been demonstrated by existing research to possess a variety of health-promoting effects, such as balancing intestinal microbiota, reducing blood cholesterol levels, enhancing immunity, and improving oral health, among others [20,21]. As shown in Figure 5, during the storage period, the total colony count of *Streptococcus thermophilus* in yogurt with a 0.4% addition level of asparagus aqueous extract was significantly higher than that of the blank control group (*p* < 0.05), indicating that a high concentration of asparagus aqueous extract most effectively promotes the growth of *Streptococcus thermophilus*. During days 7–28 of storage, the total colony count of *Lactobacillus bulgaricus* in yogurt with a 0.2% addition level was significantly higher than that of the blank control group (*p* < 0.05), suggesting that a 0.2% addition level is more suitable for promoting the growth of *Lactobacillus bulgaricus*. Changes in physicochemical indicators, such as total acidity, can promote the growth of certain specific microorganisms [22].

### 2.6. Changes in Texture Properties of Asparagus Yogurt During Storage

Texture is an important aspect of yogurt quality, playing a significant role in sensory evaluation and consumer acceptability. the texture properties of set-style yogurt, a high hardness indicates a strong shaping ability of the yogurt, enabling it to maintain a more stable overall form [23]. During the storage period, the hardness of each group exhibited a trend of initially increasing and then decreasing with storage time. As can be seen from Table 1, on the 14th day, the hardness of all groups reached its maximum value. This suggests that as the addition level of asparagus aqueous extract increases, the asparagus yogurt demonstrates greater stability during storage compared to the control yogurt, and is better able to preserve the overall structure of set-style yogurt.

Adhesiveness Index: It reflects the energy dissipation of the probe due to the viscous effects exerted by the measured sample [24]. As the storage time increased, the adhesiveness indices of all experimental groups were significantly superior to those of the blank control group (*p* < 0.05). Furthermore, a positive correlation was observed between the adhesiveness index and the increasing addition level of asparagus aqueous extract. This indicates that the asparagus aqueous extract has an enhancing effect on the adhesiveness index of yogurt, resulting in stronger adhesion capabilities of the yogurt and an improvement in product quality.

Cohesiveness: It represents the counteracting force that a test sample exhibits against a second extrusion after undergoing an initial extrusion deformation, with a higher value indicating greater system stability [25]. During the storage period, the cohesiveness of the experimental groups (with addition levels of 0.1%, 0.2%, and 0.4%) was significantly higher than that of the blank control group (*p* < 0.05), suggesting that the asparagus aqueous extract can enhance the cohesiveness of yogurt. Moreover, the cohesiveness of yogurt with a 0.4% addition level was significantly higher than that of the other groups throughout the storage period (*p* < 0.05), indicating that a high concentration of asparagus aqueous extract is more effective in improving the cohesiveness of yogurt.

As can be seen from Table 1, with the increase in the addition level of asparagus aqueous extract, the consistency of yogurt also rises accordingly. Moreover, the consistency of yogurt with a 0.4% addition level is significantly higher than that of other groups during the storage period (*p* < 0.05). This indicates that asparagus aqueous extract can effectively enhance the consistency of yogurt, which is more conducive to the development of set-style yogurt.

### 2.7. Chroma Changes in Yogurt During Storage

As indicated in the table, during the storage period, no significant changes were observed in the L and a values, suggesting that the asparagus aqueous extract has no significant impact on these parameters (*p* > 0.05). According to the Table 2, as the addition level of asparagus aqueous extract increases, the b value of the yogurt also rises, while the L and a values remain stable. This demonstrates that the asparagus aqueous extract exerts no significant influence on the L and a values of yogurt (*p* > 0.05), but has a significant effect on its b value (*p* < 0.05).

### 2.8. Changes in the Antioxidant Capacity of Asparagus Yogurt During the Storage Period

**Figure 6:**
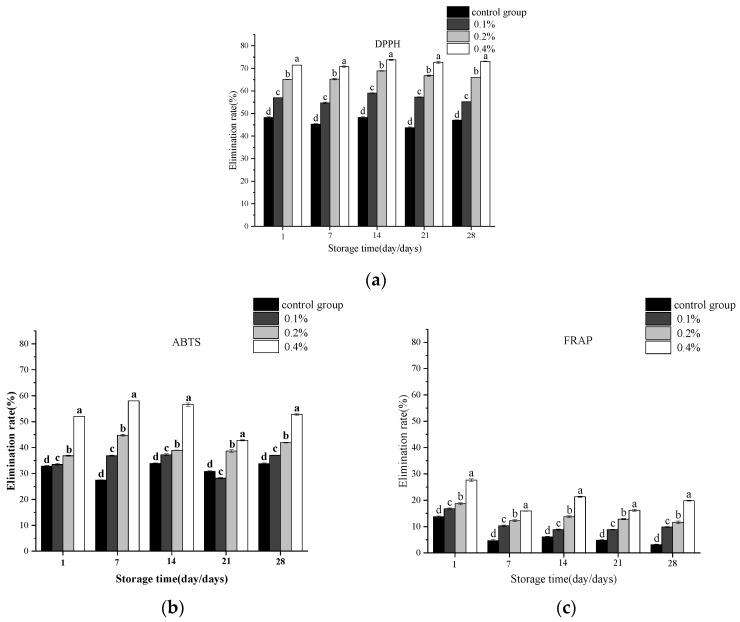
Changes in (**a**) DPPH, (**b**) ABTSA, and (**c**) FRAPof yogurts during storage. Note: Different lowercase letters indicate significant differences (*p* < 0.05).

As can be seen from Figure 6, with the increasing addition levels of asparagus aqueous extract, the antioxidant capacities of yogurt samples with 0.1%, 0.2%, and 0.4% extract additions were all significantly higher than those of the blank control group. This indicates that a greater addition of asparagus aqueous extract leads to enhanced antioxidant capacity in yogurt. Furthermore, the incorporation of asparagus aqueous extract significantly improved the free radical scavenging abilities of yogurt as assessed by DPPH, FRAP, and ABTS assays. This outcome may be attributed to the polyphenols present in the asparagus extract. Notably, the yogurt with a 0.4% addition level exhibited an ABTS free radical scavenging rate as high as 58.4% on day 7, which was significantly superior to those of the other experimental groups and the blank control group (*p* < 0.05). Asparagus extract is rich in bioactive substances such as polyphenols, flavonoids, flavanols, tannins, and ascorbic acid, which can significantly enhance the body’s antioxidant capacity [26].

### 2.9. Changes in the Volatile Components in Yogurt During the Storage Period

**Figure 7:**
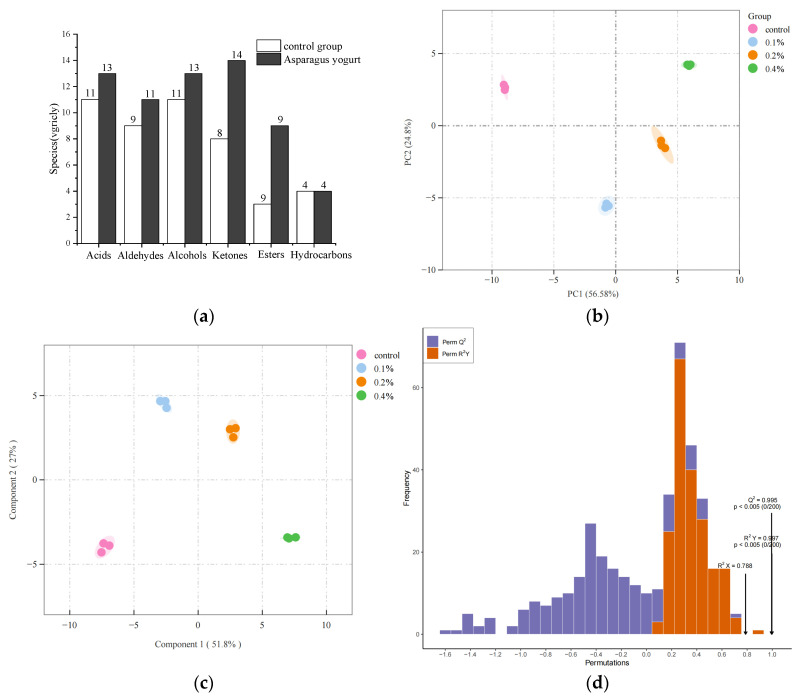
(**a**) Volatile component category quantities across different yogurt fermentation groups, (**b**) PCA plot, (**c**) OPLS-DA plot, and (**d**) model validation plot.

Aroma is a relevant indicator for evaluating food quality, which greatly influences consumers’ preferences and acceptance. The unique aroma of fermented yogurt is provided by volatile compounds. The aroma of yogurt is affected by various processes, raw materials, and other factors [27]. As can be seen from Figure 7a, using GC-MS technology, a total of 64 volatile flavor substances were detected in Yuanjiang *Miscanthus lutarioriparius* yogurt, including 13 acids (20.31%), 11 aldehydes (17.19%), 13 alcohols (20.31%), 14 ketones (21.88%), 9 esters (14.06%), and 4 hydrocarbons (6.25%). In contrast, 45 volatile flavor substances were identified in the blank control group yogurt, consisting of 11 acids (24.44%), 8 ketones (17.78%), 9 aldehydes (20%), 11 alcohols (24.44%), 3 esters (6.67%), and 4 hydrocarbons (8.89%).

Compared with the blank control group, the contents of alcohols, esters, ketones, and aldehydes in the volatile components of Yuanjiang *Miscanthus lutarioriparius* yogurt were all higher, while the contents of acids and hydrocarbons were lower. These results indicate that Yuanjiang *Miscanthus lutarioriparius* yogurt has a better flavor, with a unique spicy aroma, and its sour taste is milder than that of ordinary yogurt, thereby endowing it with superior flavor characteristics.

To further visualize the differences between the control group and yogurt groups with different addition levels of asparagus aqueous extract, principal component analysis (PCA) was performed. As shown in Figure 7b, the first two principal components collectively explained 78.8% of the total variability (with PC1 contributing 51.8% and PC2 contributing 27.0%), indicating that the model has good explanatory power. The sample points of the four treatment groups showed significant separation in the spatial distribution. The control group was concentrated on the far right of the figure, which was distinctly different from the other treatment groups. This suggests that the control group samples have a unique volatile substance profile, which is obviously different from all the added groups. Meanwhile, the addition of asparagus aqueous extract significantly altered the compositional characteristics of volatile compounds in yogurt, and the degree of influence was closely related to the addition concentration.

To further distinguish the differences between different treatment groups, orthogonal partial least squares-discriminant analysis (OPLS-DA) was used to analyze the differences in volatile substances among different treatment groups. As shown in Figure 7c, there was obvious separation between different sample groups, and cross-validation analysis confirmed the reliability of the model (R2Y = 0.997, Q2 = 0.995; Figure 7c). Based on the variable importance in projection (VIP > 1) from the OPLS-DA model, 38 volatile compounds were identified as key differential flavor compounds among the identified compounds. These 38 compounds included 9 ketones, 7 acids, 7 aldehydes, 6 esters, 6 aldehydes, and 3 hydrocarbons.

To visualize the differences in volatile compounds among different groups, cluster heatmap analysis was further performed to analyze the composition and content of volatile compounds in different samples. As shown in Figure 7d, obvious differences in distribution were observed among different samples. Compounds **1**–**20** were clustered together and considered as the characteristic volatile components of the control group. Cluster analysis showed that, compared with the control group, the content of volatile substances in the experimental groups gradually increased with the increase in the content of asparagus aqueous extract for compounds **21**–**64**.

**Figure 8:**
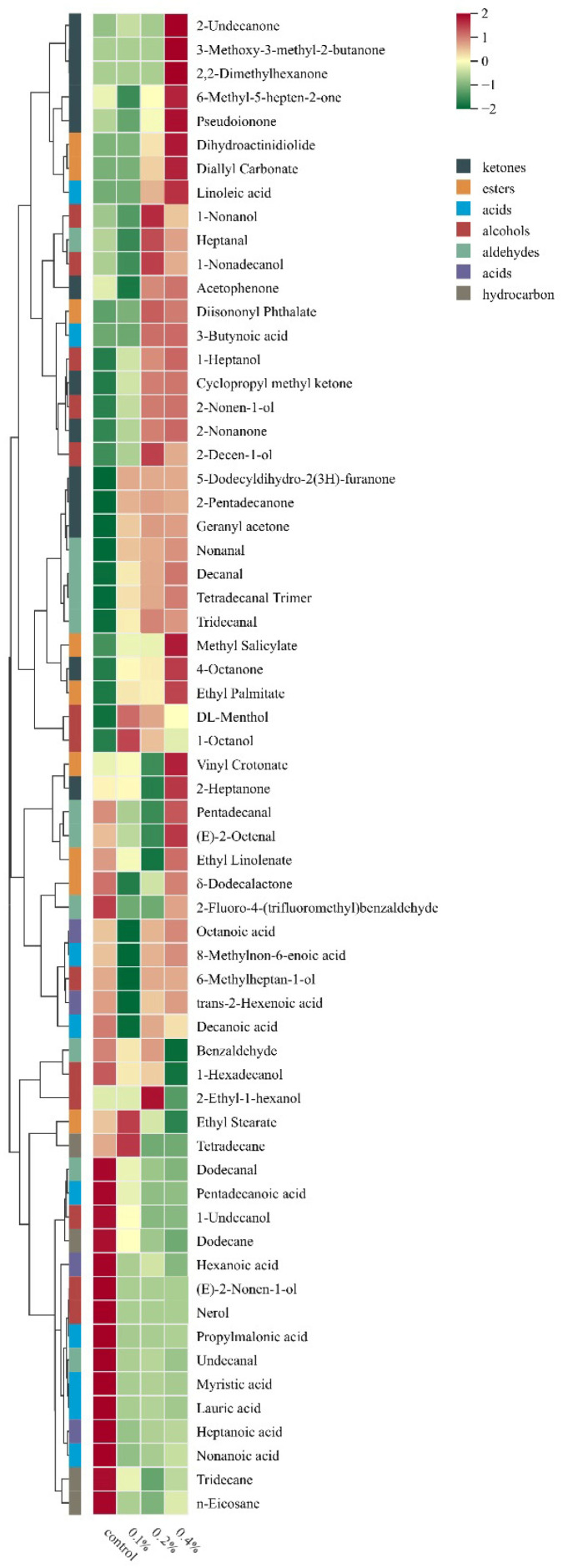
Clustering heatmap of the differential volatile compounds. Color represents the concentration of the compounds, with green and red suggesting low concentration and high concentration, respectively. The darker color indicates the higher concentration of the compounds.

As shown in Figure 8, alcohols generally have a relatively high taste threshold, thus exerting little negative impact on the overall taste of yogurt [28]. The alcohol contents in the four types of yogurt were as follows: 4.28% in the blank control group, 5.46% in the 0.1% addition group, 7.33% in the 0.2% addition group, and 7.52% in the 0.4% addition group. Due to their relatively low contents compared with other substances, these two types of yogurt (0.2% and 0.4% addition groups) were not significantly affected.

Acids are the most important part of the flavor composition of yogurt and play a vital role in the taste and aroma of yogurt [27]. There was a significant difference in the acid content between the blank control group and the asparagus yogurt groups: 35.14% in the blank control group, 19.96% in the 0.1% addition group, 14.22% in the 0.2% addition group, and 25% in the 0.4% addition group.

The substances commonly detected in all four types of yogurt included caprylic acid, nonanoic acid, decanoic acid, lauric acid, and myristic acid, whose presence contributes to the sour and refreshing taste of yogurt. Specifically, caprylic acid has a vegetable-like aroma; nonanoic acid presents a coconut-like fragrance with a faint animal fat scent; decanoic acid enhances the milk aroma of yogurt; lauric acid has a laurel oil aroma; and myristic acid has a pungent flavor. Linoleic acid, unique to asparagus yogurt, imparts a mellow fatty aroma to it. Excessive acids in yogurt can cause negative sensory effects. The 0.1% and 0.2% addition groups had much lower acid contents than the blank control group and the 0.4% addition group, which is exactly why these two addition levels resulted in better sensory properties.

Ketones are produced by amino acid degradation and unsaturated fatty acid oxidation, and they have a significant impact on the flavor of yogurt [29]. The contents of ketones in the four types of yogurt were as follows: 11.44% in the blank control group, 18.67% in the 0.1% addition group, 22.93% in the 0.2% addition group, and 27.92% in the 0.4% addition group. The ketones commonly detected that contribute to yogurt flavor include acetophenone, 2-nonanone, 2-heptanone, 6-methyl-5-hepten-2-one, methyl nonyl ketone, and 6,10-dimethyl-5,9-undecadien-2-one. Specifically, acetophenone has a hawthorn-like aroma; 2-nonanone exhibits an aroma similar to that of fruits, flowers, animal fats, and herbs; 2-heptanone has a pear-like fruity fragrance; 6-methyl-5-hepten-2-one presents a citrus aroma; methyl nonyl ketone has a fresh floral scent; and 6,10-dimethyl-5,9-undecadien-2-one has a unique rue-like aroma.

Aldehyde compounds are one of the main flavor components of yogurt, with special and diverse aromas. They generally have low flavor thresholds and exert a significant impact on human perception of flavor [30]. The contents of aldehydes in the four groups of yogurt were 11.36% in the blank control group, 13.16% in the 0.1% addition group, 13.7% in the 0.2% addition group, and 14.1% in the 0.4% addition group. The aldehydes commonly detected in all four groups that have a relatively large impact on yogurt flavor include heptanal, trans-2-octenal, nonanal, decanal, and undecanal. Heptanal has a fruity aroma; trans-2-octenal presents a fatty and meaty aroma with cucumber and chicken notes; nonanal has a rose and citrus-like fragrance; decanal gives yogurt a sweet orange and tangerine flavor with a fatty undertone; and undecanal imparts a faint sweet taste to yogurt.

Asparagus yogurt contains two unique aldehydes that enhance its distinct flavor: tridecanal trimer, which has a mild fatty taste with slight citrus and iris-like notes; and tridecanal, which has a sweet orange and grapefruit-like flavor accompanied by waxy and oily undertones. This also indicates that the asparagus aqueous extract promotes the production of favorable flavor substances during yogurt fermentation, resulting in a unique product flavor.

Ester compounds are generally formed through the hydrolysis of acids and alcohols, as well as bacterial metabolism [31]. Although their content is low, they have low thresholds and thus make a significant contribution to the flavor of yogurt. Compared with the blank control yogurt, asparagus yogurt had a higher content of ester compounds, which may be attributed to the promotion of probiotic growth during fermentation by the asparagus extract. The contents of esters were 6.25% in the blank control group, 8.81% in the 0.1% addition group, 11.54% in the 0.2% addition group, and 17.63% in the 0.4% addition group. The commonly detected ester contributing to yogurt flavor is δ-dodecalactone, which gives yogurt a coconut-like aroma.

Asparagus yogurt contains unique flavor substances such as methyl salicylate and ethyl palmitate. Methyl salicylate imparts a characteristic wintergreen odor to yogurt, while ethyl palmitate has a faint waxy fragrance with fruity and creamy notes. This indicates that unique flavors are generated during the fermentation of asparagus yogurt, distinguishing it from other yogurts.

Asparagus yogurt contains unique ketones such as geranyl acetone, cyclopropyl methyl ketone, 4-octanone, and 2-pentadecanone, which impart a penetrating fresh and sweet fragrance, a slight rose scent, and a mild resinous aroma. Among them, the 0.4% addition group had higher contents of these components than other groups, indicating that the asparagus aqueous extract can promote the production of such aromatic substances during yogurt fermentation, thereby improving the flavor of yogurt.

Hydrocarbons have high flavor thresholds in yogurt and have no impact on flavor [32]. Their contents in the four groups of yogurt were 4.61% in the blank control group, 4.45% in the 0.1% addition group, 4.33% in the 0.2% addition group, and 4.36% in the 0.4% addition group.

### 2.10. Sensory Evaluation Score of Yogurt

**Figure 9:**
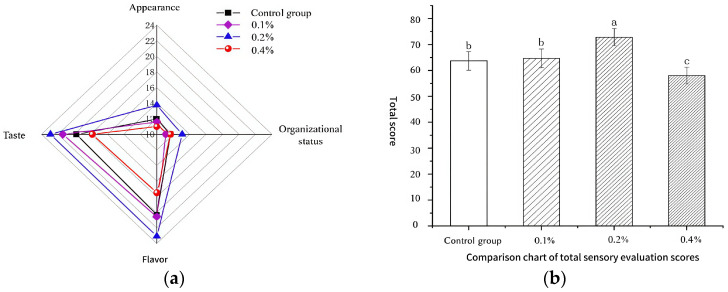
(**a**) Radar chart of sensory score and (**b**) total sensory score. Note: Different lowercase letters indicate significant differences (*p* < 0.05).

The sensory quality of yogurt plays a crucial role in its flavor characteristics and consumer acceptance [33]. As illustrated in Figure 9a, the yogurt samples fortified with 0.1% and 0.2% asparagus aqueous extract demonstrated significant improvements in taste, texture, and appearance, indicating that the incorporation of low-concentration asparagus aqueous extract can effectively enhance the sensory attributes of yogurt, thereby providing consumers with a superior consumption experience. However, the yogurt sample with a 0.4% addition level received notably low scores in terms of flavor, appearance, and mouthfeel, likely attributable to an excessive amount of asparagus aqueous extract, which resulted in an overly yellow hue, excessive acidity, and an overpowering asparagus flavor, thereby negatively impacting the overall sensory evaluation.

As depicted in Figure 9b, the sensory score of the yogurt sample with a 0.2% addition level was significantly higher than that of the blank control group (*p* < 0.05). This suggests that a 0.2% addition level of asparagus aqueous extract can more effectively improve the sensory attributes of yogurt, leading to substantial enhancements in mouthfeel, flavor, texture, and appearance. In contrast, no significant difference was observed between the yogurt sample with a 0.1% addition level and the blank control group (*p* > 0.05), indicating that a 0.1% addition level has limited overall improvement on yogurt. Nevertheless, as shown in Figure 9a, the mouthfeel of the yogurt sample with a 0.1% addition level was superior to that of the blank control group, suggesting that an appropriate amount of asparagus aqueous extract can contribute to a better mouthfeel in yogurt. The overall sensory score of the yogurt sample with a 0.4% addition level was also significantly lower than those of the other three groups (*p* < 0.05), further confirming that an excessive addition of 0.4% asparagus aqueous extract leads to an unsatisfactory sensory experience in yogurt.

## 3. Materials and Methods

### 3.1. Material

Yuanjiang *Miscanthus lutarioriparius* powder (provided by Hunan Boda Tianneng Co., Ltd., Huzhou, China); lactic acid bacteria starter (purchased from Beijing Chuanxiu Technology Co., Ltd., Beijing, China); skimmed milk powder (supplied by Fonterra Trading Co., Ltd., Shanghai, China); sucrose (commercially available); sodium hydroxide and sodium chloride (obtained from Sinopharm Chemical Reagent Co., Ltd., Beijing, China).

### 3.2. Test Method

#### 3.2.1. Preparation of Yogurt with Aqueous Extract of Yuanjiang Asparagus

The extraction process was performed with modifications based on the method described by He et al. [34]. Specifically, Yuanjiang *Miscanthus lutarioriparius* powder was mixed with water at a ratio of 1:90 (g:mL), followed by extraction in a water bath at 90 °C for 4 h.

The method described by Tang et al. [35]. was adopted with modifications. Specifically, 12% reconstituted skim milk was prepared, to which Yuanjiang *Miscanthus lutarioriparius* aqueous extract was added at final concentrations (m/v) of 0%, 0.1%, 0.2%, and 0.4%, respectively. The mixtures were thoroughly blended and preheated to 60–75 °C.

The formulated reconstituted skim milk samples were subjected to high-speed shearing using a high-shear mixer at 20,000 r/min for 2 min, with this shearing process repeated three times. The homogenized reconstituted skim milk was then pasteurized at 90–95 °C for 10 min.

Each sample group was inoculated with a lactic acid bacteria starter at an inoculum size of 2%. The inoculated reconstituted skim milk was incubated at 43 °C until curdling, which was defined as the end of fermentation. The curdled fermented milk was subsequently transferred to a 4 °C refrigerator for cold storage and maturation.

#### 3.2.2. Storage Period

Using plain yogurt without asparagus aqueous extract as the blank control group, and yogurts with asparagus aqueous extract added at 0.1%, 0.2%, and 0.4% as experimental groups, parameters including acidity, viscosity, water holding capacity, viable bacterial counts, color, texture, and rheological properties were determined during the 28-day storage period (from day 1 to day 28).

#### 3.2.3. Physicochemical Analysis

Determination of Acidity (Based on Phenolphthalein Indicator Method in GB 5009.239-2016 [36]). The viscosity of yogurt was measured using a rotational viscometer. DPPH/ABTS Radical Scavenging: Methods described by Li et al. [37] and Guo et al. [38]. FRAP Assay: Performed using a commercial kit (Solarbio, Beijing, China).

#### 3.2.4. Water Holding Capacity

Approximately 10 g of fermented milk sample (accurately weighed) was centrifuged at 6000× *g* and 10 °C for 30 min. The supernatant was discarded, and the tube was inverted for 10 min to allow complete drainage. The mass of the precipitate was immediately recorded. WHC was calculated as follows:

WHC (%) = [(m_2_ − m_0_) / (m_1_ − m_0_)] × 100 (1)

m_0_: Mass of centrifuge tube (g); m_1_: Mass of centrifuge tube + sample before centrifugation (g); m_2_: Mass of centrifuge tube + precipitate after centrifugation (g).

#### 3.2.5. Determination of Rheological Properties of Yogurt

The method described by Fei Gao et al. [39] was referenced. A rotational rheometer was used with a PP50 mm tray and a flat-plate fixture (40 mm in diameter), and the gap between the plate and tray was 1 mm. Static sweep was performed on the fermented milk at 25 °C, with the apparent viscosity measured at a shear rate ranging from 0.01 to 100 s^−1^. At the same temperature, stress sweep was conducted on the fermented milk at a frequency of 1 Hz, a shear rate of 1 s^−1^, and a strain ranging from 0.01% to 10% to determine the storage modulus (G′) and loss modulus (G″). At the same temperature, frequency sweep was carried out on the fermented milk at a strain of 1% and a frequency ranging from 0.1 to 10.0 Hz to determine the G′ and G″ of the fermented milk.

#### 3.2.6. Determination of Total Bacterial Colonies

The viable count of lactic acid bacteria was determined in accordance with GB 4789.35-2023 [40] National Food Safety Standard—Microbiological Examination of Foods—Examination of Lactic Acid Bacteria.

#### 3.2.7. Determination of Hardness, Consistency, Adhesiveness Index, and Cohesiveness

The measurements were performed using a texture analyzer with the following parameter settings: A/BE probe (35 mm in diameter); pre-test speed: 1 mm/s; test speed: 1 mm/s; post-test speed: 10 mm/s; probe penetration depth into the sample: 30 mm; trigger type: automatic; applied force: 5 g.

#### 3.2.8. Determination of Chromaticity

A colorimeter was used for the determination, measuring the brightness (L* value), red-green index (a* value), and yellow-blue index (b* value). The total color difference ΔE* was calculated using the formula:

ΔE* = (Δa*^2^ + Δb*^2^ + ΔL*^2^)^1/2^ (2)

A color change with ΔE* > 2 is easily perceptible to the human eye [41].

#### 3.2.9. Determination of Volatile Components

The method described by Fei Bakırcı İ et al. [42] and B.J. Wright et al. [43] was referenced. To determine the volatile components of asparagus yogurt, samples were taken from the blank control group yogurt and the experimental groups (with asparagus aqueous extract addition amounts of 0.1%, 0.2%, and 0.4%) after 24 h of post-ripening, followed by GC-MS detection and analysis.

Specifically, 5 g of yogurt sample was weighed and placed in a 20 mL extraction vial, followed by the addition of 2 g of sodium chloride and 5 mL of distilled water. The sample was then thoroughly mixed with water and sodium chloride, and placed in a 45 °C constant-temperature water bath for salting-out for 30 min. The aged extraction head of the solid-phase microextraction (SPME) device was inserted into the extraction vial, with the extraction head being approximately 1 cm away from the sample for headspace extraction, and the salting-out extraction was performed for about 40 min. The extraction head, which had completed extraction and adsorption, was removed and inserted into the injection port of the GC-MS instrument. The fiber head was pushed out and desorbed at 250 °C for 6 min, followed by GC-MS analysis.

A programmed temperature rise method was adopted: the initial temperature was 40 °C, held for 3 min, then increased to 180 °C at a heating rate of 5 °C/min and held for 2 min, then further increased to 250 °C at 10 °C and held for 5 min; the injection port temperature was 250 °C; the carrier gas was helium with a flow rate of 1 mL/min; the transfer line temperature was 230 °C; and splitless injection was used.

Electron ionization (EI) was used as the ionization mode, with an electron energy of 70 eV; the ion source temperature was 250 °C, and the mass scanning range was 35–400 amu; the emission current was 100 μA, and the detection voltage was 1.4 Kv.

The NIST2011 standard chromatographic database was used to manually search for the corresponding experimental spectrum data. The experimental results were corrected by referring to relevant literature and then quantitatively analyzed according to the storage date of the samples. The relative percentage concentration of the products was estimated based on the proportion of the single peak area.

#### 3.2.10. Sensory Evaluation

The sensory evaluation employed in this study received approval from the Hunan Agricultural University Institutional Review Board (HAU-IRB). With reference to the sensory requirements of GB 19302-2010 National Food Safety Standard for Fermented Milk and the sensory evaluation rules of RHB 104-2020 Yoghurt Sensory Evaluation Guidelines, a sensory evaluation panel consisting of 20 professionally trained assessors (ten females and ten males, aged 22–42) was selected to evaluate the appearance, texture, flavor, and taste of the fermented milk. Sensory evaluation form is shown in Table 3:

#### 3.2.11. Determination of Storage Quality of Asparagus Aqueous Extract Yogurt

Yogurt samples with different concentrations of asparagus aqueous extract added were used as the experimental groups, and yogurt without asparagus aqueous extract added was used as the control group. The changes in physicochemical properties and antioxidant activity of asparagus yogurt during storage were studied.

#### 3.2.12. Data Processing

The experimental data were statistically processed using Microsoft Excel 2010, and one-way analysis of variance (ANOVA) was performed using SPSS 26.0. Graphs were plotted with Origin 2021 software. Each experiment was repeated 3 times, and the results were presented as mean ± standard deviation.

## 4. Conclusions

This study investigated the physicochemical quality, changes in antioxidant capacity, volatile components, main flavor substances, and sensory properties of Yuanjiang *Miscanthus lutarioriparius* yogurt with different additive amounts during storage. The results showed that Yuanjiang *Miscanthus lutarioriparius* water extract, as a functional additive, can effectively improve the physical stability of yogurt during storage, inhibit post-acidification, promote the proliferation of probiotics, significantly enhance antioxidant activity, and optimize its color and texture. Yuanjiang *Miscanthus lutarioriparius* yogurt has a better flavor, with a unique spicy aroma, and its sour taste is milder than that of ordinary yogurt. Among the tested groups, the 0.2% addition level exhibited the best performance in inhibiting post-acidification.

This study provides a theoretical basis for the development of functional Yuanjiang *Miscanthus lutarioriparius* yogurt with enhanced stability and health benefits (such as antioxidant activity and probiotic activity).

## Figures and Tables

**Table 1:** Changes in texture of yogurts during storage.

		Control Group	0.1%	0.2%	0.4%
Hardness g	1	43.90 ± 0.23 d	55.09 ± 0.37 c	58.38 ± 0.47 b	61.08 ± 0.31 a
	7	72.57 ± 0.46 d	87.78 ± 0.26 c	95.06 ± 0.56 b	104.13 ± 0.68 a
	14	62.06 ± 0.54 c	91.63 ± 0.55 b	95.67 ± 0.93 b	168.85 ± 0.37 a
	21	40.92 ± 1.01 b	39.87 ± 1.26 b	43.09 ± 0.50 a	44.76 ± 0.20 a
Consistency g·s	1	123.4 ± 0.34 d	144.79 ± 0.60 c	152.51 ± 0.38 b	184.78 ± 0.44 a
	7	356.53 ± 2.88 d	455.65 ± 2.68 c	488.29 ± 2.12 b	515.06 ± 4.72 a
	14	431.65 ± 2.34 d	470.43 ± 1.21 c	512.55 ± 4.14 b	541.94 ± 3.55 a
	21	441.02 ± 2.71 d	481.96 ± 1.17 c	535.61 ± 2.41 b	553.52 ± 2.14 a
Cohesion g	1	97.00 ± 0.51 d	106.15 ± 0.45 c	108.69 ± 0.45 a	107.40 ± 0.43 b
	7	157.11 ± 1.49 d	168.93 ± 0.53 c	172.36 ± 1.67 b	218.98 ± 1.19 a
	14	124.63 ± 0.71 c	117.96 ± 0.87 d	131.72 ± 0.88 b	142.87 ± 1.67 a
	21	94.24 ± 1.26 c	98.10 ± 1.31 b	105.51 ± 0.81 a	98.72 ± 0.65 b
Viscosity index	1	23.50 ± 0.37 b	24.00 ± 0.12 b	24.23 ± 0.57 b	25.65 ± 0.40 a
	7	66.39 ± 0.80 d	69.56 ± 0.22 c	76.46 ± 1.73 b	86.27 ± 0.66 a
	14	23.34 ± 2.18 c	19.33 ± 0.98 d	37.91 ± 1.31 b	46.18 ± 1.04 a
	21	14.81 ± 0.61 b	15.53 ± 0.27 ab	16.27 ± 0.74 a	16.28 ± 0.26 a

Note: Different lowercase letters in the same row indicate significant differences (*p* < 0.05).

**Table 2:** Changes in color values of yogurts during storage.

	Storage Time	Control Group	0.1%	0.2%	0.4%
L	1	71.90 ± 0.51 a	71.57 ± 0.25 a	70.93 ± 1.17 a	70.48 ± 0.35 a
	7	71.88 ± 0.27 a	71.17 ± 0.31 ab	70.40 ± 0.72 b	70.26 ± 0.23 b
	14	71.85 ± 0.73 a	71.21 ± 0.48 ab	68.64 ± 0.43 bc	68.02 ± 2.02 c
	21	71.08 ± 0.41 ab	72.31 ± 0.42 a	70.96 ± 0.65 ab	69.84 ± 0.97 b
	28	70.36 ± 0.81 a	72.08 ± 0.52 a	70.76 ± 0.57 a	70.16 ± 1.53 a
a	1	−2.48 ± 0.03 a	−2.46 ± 0.16 a	−2.57 ± 0.29 a	−2.45 ± 0.14 a
	7	−2.47 ± 0.06 a	−2.31 ± 0.11 a	−2.44 ± 0.11 a	−2.30 ± 0.07 a
	14	−2.50 ± 0.12 b	−2.43 ± 0.13 a	−2.08 ± 0.05 b	−2.28 ± 0.16 ab
	21	−2.37 ± 0.06 a	−2.68 ± 0.06 b	−2.49 ± 0.11 ab	−2.37 ± 0.09 a
	28	−2.39 ± 0.17 a	−2.55 ± 0.08 a	−2.65 ± 0.24 a	−2.35 ± 0.07 a
b	1	1.03 ± 0.42 c	1.75 ± 0.60 c	4.1 ± 0.55 b	3.48 ± 0.93 b
	7	0.94 ± 0.43 b	1.42 ± 0.53 b	4.04 ± 0.52 a	3.51 ± 0.89 a
	14	1.49 ± 0.48 c	2.01 ± 0.67 bc	3.85 ± 0.11 ab	4.67 ± 1.56 a
	21	1.23 ± 0.31 c	3.41 ± 0.51 b	3.79 ± 0.60 b	5.51 ± 0.83 a
	28	1.50 ± 0.37 c	3.54 ± 0.62 b	4.94 ± 0.06 a	5.81 ± 0.43 a

Note: Different lowercase letters in the same row indicate significant differences (*p* < 0.05), “L” represents lightness, “a” represents the red-green axis, and “b” represents the yellow-blue axis in the CIELAB color space.

**Table 3:** Sensory evaluation table.

Project	Appearance	Organizational Status	Flavor	Taste
	20 points	20 points	30 points	30 points
1	Uniform in color, without lumps or whey separation 12–20	The curd is uniform and fine, with a smooth surface free of bubbles or only a few bubbles. 12–20	Have a yogurt flavor, a moderate asparagus aroma, and is highly acceptable with no other off-flavors 20–30	The yogurt is moderately sweet and sour, with a moderate asparagus flavor. It has a delicate and thick texture 20–30
2	The uniformity of color is average, with a few lumps and a small amount of whey precipitated 6–11	The curd is relatively uniform, with a rough surface and a few bubbles 6–11	Have a yogurt flavor, but the asparagus smell is either too strong or too weak, with a slight off-flavor, which is acceptable 10–19	The yogurt is slightly sour or sweet, and the asparagus has a stronger or lighter flavor with a slight frosty texture 10–19
3	The color uniformity is poor, with severe lumps and serious whey precipitation < 6	There is no curd and it is in a paste-like state < 6	Do not have the inherent flavor of yogurt, and the asparagus smell is too strong or absent. It has a strong off-flavor and is unacceptable < 10	The sourness of yogurt is unacceptable. The frosted texture is severe, and the asparagus flavor is too strong or absent < 10

## Data Availability

The data presented in this study are available on request from the corresponding author.

## References

[1] Kazou M., Grafakou A., Tsakalidou E., Georgalaki M. (2021). Zooming Into the Microbiota of Home-Made and Industrial Kefir Produced in Greece Using Classical Microbiological and Amplicon-Based Metagenomics Analyses. Front. Microbiol..

[2] Asefa Z., Tesfaye A., Desalegn A., Daba T., Haile T. (2025). Formulation and Evaluation of Probiotic Starter Culture: Impact on Ethiopian Cottage Cheese “Ayib” Safety, Stability, Sensory Acceptability and Antioxidant Potential. One Health Outlook.

[3] Pires de Oliveira Galdino I.K.C., da Silva M.O.M., da Silva A.P.A., Santos V.N., Feitosa R.L.P., Ferreira L.C.N., Dantas G.C., Dos Santos Pereira E.V., de Oliveira T.A., Dos Santos K.M.O. (2023). β-Glucosidase Activity and Antimicrobial Properties of Potentially Probiotic Autochthonous Lactic Cultures. PeerJ.

[4] Babio N., Mena-Sánchez G., Salas-Salvadó J. (2017). Eyond the nutritional value of yogurt: A diet quality indicator?. Nutr. Hosp..

[5] Martins N., Oliveira M.B.P.P., Ferreira I.C.F.R. (2018). Development of Functional Dairy Foods. Bioactive Molecules in Food.

[6] Sabaragamuwa R., Perera C.O. (2023). Total Triterpenes, Polyphenols, Flavonoids, and Antioxidant Activity of Bioactive Phytochemicals of Centella Asiatica by Different Extraction Techniques. Foods.

[7] Cao Q., Yan J., Sun Z., Gong L., Wu H., Tan S., Lei Y., Jiang B., Wang Y. (2021). Simultaneous Optimization of Ultrasound-Assisted Extraction for Total Flavonoid Content and Antioxidant Activity of the Tender Stem of Triarrhena Lutarioriparia Using Response Surface Methodology. Food Sci. Biotechnol..

[8] Mondal S., Rahaman S.T. (2020). Flavonoids: A Vital Resource in Healthcare and Medicine. Pharm. Pharmacol. Int. J..

[9] Matos J., Afonso C., Cardoso C., Serralheiro M.L., Bandarra N.M. (2021). Yogurt Enriched with Isochrysis Galbana: An Innovative Functional Food. Foods.

[10] Duan W., Guan Q., Zhang H.-L., Wang F.-Z., Lu R., Li D.-M., Geng Y., Xu Z.-H. (2023). Improving Flavor, Bioactivity, and Changing Metabolic Profiles of Goji Juice by Selected Lactic Acid Bacteria Fermentation. Food Chem..

[11] Fan X., Liu M., Shi Z., Zhang T., Du L., Wu Z., Zeng X., Wu X., Pan D. (2024). Binary Probiotic Fermentation Promotes Signal (Cyclic AMP) Exchange to Increases the Number of Viable Probiotics, Anthocyanins and Polyphenol Content, and the Odor Scores of Wolfberry Fermented Beverages. Food Chem..

[12] Zhang L., Jiang L., Yan W., Tao H., Yao C., An L., Sun Y., Hu T., Sun W., Qian X. (2025). Exogenous Additives Reshape the Microbiome and Promote the Reduction of Resistome in Co-Composting of Pig Manure and Mushroom Residue. J. Hazard. Mater..

[13] Lee W.J., Lucey J.A. (2004). Structure and Physical Properties of Yogurt Gels: Effect of Inoculation Rate and Incubation Temperature. J. Dairy Sci..

[14] Nanakali N.M., Muhammad Al-saadi J., Sulaiman Hadi C. (2023). Functional and Physiochemical Properties of the Yoghurt Modified by Heat Lactosylation and Microbial Transglutaminase Cross-Linking of Milk Proteins. Food Sci. Nutr..

[15] Zhang X., LaPointe G., Liu Y., Wang X., Xiao L., Zhao X., Li W. (2023). Comparative Analysis of Exopolysaccharide-Producing Lactiplantibacillus Plantarum with Ropy and Non-Ropy Phenotypes on the Gel Properties and Protein Conformation of Fermented Milk. Food Chem..

[16] Kong X., Xiao Z., Du M., Wang K., Yu W., Chen Y., Liu Z., Cheng Y., Gan J. (2022). Physicochemical, Textural, and Sensorial Properties of Soy Yogurt as Affected by Addition of Low Acyl Gellan Gum. Gels.

[17] Riantiningtyas R.R., Sager V.F., Chow C.Y., Thybo C.D., Bredie W.L.P., Ahrné L. (2021). 3D Printing of a High Protein Yoghurt-Based Gel: Effect of Protein Enrichment and Gelatine on Physical and Sensory Properties. Food Res. Int..

[18] Gonçalves R.F.S., Rodrigues R., Vicente A.A., Pinheiro A.C. (2023). Incorporation of Solid Lipid Nanoparticles into Stirred Yogurt: Effects in Physicochemical and Rheological Properties during Shelf-Life. Nanomaterials.

[19] Bianco S., Panja S., Adams D.J. (2022). Using Rheology to Understand Transient and Dynamic Gels. Gels.

[20] Sharifi-Rad J., Rodrigues C.F., Stojanović-Radić Z., Dimitrijević M., Aleksić A., Neffe-Skocińska K., Zielińska D., Kołożyn-Krajewska D., Salehi B., Milton Prabu S. (2020). Probiotics: Versatile Bioactive Components in Promoting Human Health. Med. Kaunas Lith..

[21] Zaib S., Hayat A., Khan I. (2024). Probiotics and Their Beneficial Health Effects. Mini Rev. Med. Chem..

[22] Xu J., Zhang T., Chen H., Dai Y., Li Z., He J., Ju R., Hou A. (2024). Study on the Fermented Grain Characteristics and Volatile Flavor Substances during the Tuqu Fermentation of Hunan Light-Flavor Baijiu. Foods.

[23] Wang L., Zhang F., Zheng B., Zhang Y., Pan L. (2023). Stability and Flavor of Set Yogurt Fortified with *Tremella Fuciformis* Polysaccharide during Cold Storage. Curr. Res. Food Sci..

[24] Liu H.-C., Urban M.W. (2021). Optical Coherence Viscometry. Appl. Phys. Lett..

[25] Lu W., Zhang Y., Xiao C., Chen D., Ye Q., Zhang C., Meng X., Wang S. (2022). The Comprehensive Utilization of Bean Dregs in High-Fiber Tofu. Foods.

[26] Chileh Chelh T., Rincon-Cervera M.A., Gomez-Mercado F., Lopez-Ruiz R., Gallon-Bedoya M., Ezzaitouni M., Guil-Guerrero J.L. (2023). Wild Asparagus Shoots Constitute a Healthy Source of Bioactive Compounds. Molecules.

[27] Zhao M., Li H., Zhang D., Li J., Wen R., Ma H., Zou T., Hou Y., Song H. (2023). Variation of Aroma Components of Pasteurized Yogurt with Different Process Combination before and after Aging by DHS/GC-O-MS. Molecules.

[28] Arslaner A. (2020). The Effects of Adding Garlic (*Allium sativum* L.) on the Volatile Composition and Quality Properties of Yogurt. Food Sci. Technol..

[29] Chi X., Yang Q., Su Y., Zhang J., Sun B., Ai N. (2024). Improvement of Rheological and Sensory Properties of *Lactobacillus helveticus* Fermented Milk by Prebiotics. Food Chem. X.

[30] Dan T., Hu H., Tian J., He B., Tai J., He Y. (2023). Influence of Different Ratios of Lactobacillus Delbrueckii Subsp. Bulgaricus and *Streptococcus Thermophilus* on Fermentation Characteristics of Yogurt. Molecules.

[31] Wu R., Yu M., Liu X., Meng L., Wang Q., Xue Y., Wu J., Yue X. (2015). Changes in Flavour and Microbial Diversity during Natural Fermentation of Suan-Cai, a Traditional Food Made in Northeast China. Int. J. Food Microbiol..

[32] Chi X., Yang Q., Su Y., Xi Y., Wang W., Sun B., Ai N. (2024). Effect of Prebiotics on Rheological Properties and Flavor Characteristics of *Streptococcus Thermophilus* Fermented Milk. Curr. Res. Food Sci..

[33] Gupta M.K., Torrico D.D., Ong L., Gras S.L., Dunshea F.R., Cottrell J.J. (2022). Plant and Dairy-Based Yogurts: A Comparison of Consumer Sensory Acceptability Linked to Textural Analysis. Foods.

[34] He T., Yu J., Wu S., Tang X., Liu C., Zhou H. (2022). Effects of Exogenous Supplementation with Yuanjiang *Miscanthus lutarioriparius* Aqueous Extract on Fermented Yogurt. China Dairy.

[35] Tang J., Tang X., Zhang F., Luo J., Liu C., Zhou H. (2023). Effect of Exogenous Addition of *Lactobacillus paracasei* LZ9077 on Properties of Set-Type Yogurt. J. Dairy Sci. Technol..

[36] (2016). National Food Safety Standard—Determination of Acidity in Food.

[37] Li G.L., Liu J., Li X., Ma L. (2021). Storage Quality of Yam and Hawthorn Yogurt and Its Antioxidation in Simulated Gastrointestinal Fluid. China Brew..

[38] Shen S., Chen D., Li X., Li T., Yuan M., Zhou Y., Ding C. (2014). Optimization of Extraction Process and Antioxidant Activity of Polysaccharides from Leaves of *Paris polyphylla*. Carbohydr. Polym..

[39] Gao F., Li D., Li H., Chen H., Mao X., Wang P. (2023). Influence of Post-Heating Treatment on the Sensory and Textural Properties of Stirred Fermented Milk. Foods.

[40] (2023). National Food Safety Standard—Food Microbiological Examination—Examination of Lactic Acid Bacteria.

[41] Bustamante M., Giménez P., Just-Borràs A., Solé-Clua I., Gombau J., Heras J.M., Sieczkowski N., Gil M., Pérez-Navarro J., Gómez-Alonso S. (2024). Use of Glutathione, Pure or as a Specific Inactivated Yeast, as an Alternative to Sulphur Dioxide for Protecting White Grape Must from Browning. Foods.

[42] Bakırcı İ., Terzioğlu M., Akkaya İ. (2023). Volatile Compounds, Antioxidant Activity, ACE Inhibitory Activity, HMF Content and Microstructure of Fruit Yoghurts. Mljekarstvo.

[43] Wright B.J., Zevchak S.E., Wright J.M., Drake M.A. (2009). The Impact of Agglomeration and Storage on Flavor and Flavor Stability of Whey Protein Concentrate 80% and Whey Protein Isolate. J. Food Sci..

